# Health related quality of life in Dutch infants, toddlers, and young children

**DOI:** 10.1186/s12955-017-0654-4

**Published:** 2017-04-24

**Authors:** S. A. Schepers, H. A. van Oers, H. Maurice-Stam, J. Huisman, C. M. Verhaak, M. A. Grootenhuis, L. Haverman

**Affiliations:** 1 St. Jude Children’s Research Hospital, Department of Psychology, Memphis, USA; 2 Emma Children’s Hospital, Academic Medical Center, Psychosocial Department, Amsterdam, The Netherlands; 3 Princess Máxima Center for pediatric oncology, Utrecht, The Netherlands; 4 Department of Medical Psychology, Amalia Children’s Hospital, Radboud University Medical Center, Nijmegen, The Netherlands

**Keywords:** Health-related quality of life, Pediatrics, Child, Psychometrics, Patient outcome assessment

## Abstract

**Background:**

The purpose of this study is to provide Dutch normative data and to assess internal consistency and known-groups validity for the TNO AZL Preschool Children Quality of Life (TAPQOL) and the acute version of the generic Pediatric Quality of Life Inventory (PedsQL 4.0) in Dutch young children aged 0–7 years.

**Methods:**

Participants were selected from a panel of a large Dutch market research agency. A sample of 794 parents (response rate 61%, 39% fathers) of children (53% boys) from the general Dutch population, completed an electronic version of the TAPQOL (*N* = 227 infants aged 0–1 years) or PedsQL 4.0 (*N* = 293 toddlers aged 2–4 years and *N* = 274 young children aged 5–7 years).

**Results:**

Except for the ‘stomach’ scale (α = .39), the TAPQOL showed acceptable to excellent internal consistency (α = .60-.88). The PedsQL 4.0 showed acceptable to excellent reliability in children aged 2–4 years (α = .60–.88) and in children aged 5–7 years (α = .76–.90). Children with a chronic health condition had lower scores than healthy children on 3 out of 12 domains of the TAPQOL (*p =* .001–.013) and on 2 out of 6 domains of the PedsQL 4.0 for children aged 2–4 years (*p =* .016–.04). The PedsQL 4.0 differentiated on all domains (*p <* .05) between children aged 5–7 years with and without a chronic health condition.

**Conclusion:**

In Dutch children aged 0–7 years old, HRQoL can be relialy measured with the TAPQOL and the PedsQL 4.0. However, it remains unclear whether these HRQoL instruments can distinguish between healthy children and children with a chronic health condition under the age of 5.

## Background

Health-Related Quality of Life (HRQoL) has become an important part of patient outcome assessment in pediatrics. A HRQoL instrument should be “multidimensional, consisting at the minimum of the physical, mental, and social health dimensions delineated by the WHO” [1]. Generic HRQoL questionnaires provide a comprehensive overview of HRQoL and allow for comparisons between healthy and disease populations [2]. Questionnaires about children’s HRQoL can be self- or proxy- reported. Self-report is usually preferred, because the child’s report is the best source of information about what he or she is experiencing [3, 4]. However, when children are too young (under the age of eight [4, 5]), too cognitively impaired, too ill or too fatigued to complete a HRQoL instrument, parent/caregiver proxy-report is recommended [6].

To reliably measure the impact of a chronic condition on children’s HRQoL compared to their healthy peers, it is important to use validated questionnaires. So far, several validated age-appropriate questionnaires to measure HRQoL in infants (0–1 years), toddlers (2–4 years), and young children (5–7 years) are available, such as the Infant and Toddler Quality Of Life questionnaire (ITQOL) [7], TNO AZL Preschool Children Quality of Life (TAPQOL) [8], the Child Health Questionnaire (CHQ) [9], and the Pediatric Quality of Life Inventory 4.0 (PedsQL) [10].

Electronic administration of measures is becoming more common and offers several advantages over pencil and paper formats. For example, data can be automatically scored and extracted, and it can be stored in the electronic medical record. The electronic questionnaires are a literal reproduction of the original paper-pencil questionnaires. Research to date suggests that data obtained by paper versus electronic rating scales are equivalent, and there are no systematic differences in outcomes with the two approaches [11]. Furthermore, patients and healthcare providers generally prefer electronic measures above paper-pencil measures [11, 12].

As part of a project on the implementation of an electronic system for the routine monitoring of patient-reported outcomes in children with a chronic disease or cancer [13, 14], electronic versions of the TAPQOL and the PedsQL 4.0 (both internationally widely used questionnaires to measure HRQoL in children) have been developed. However, for infants aged 0–1 years (TAPQOL), toddlers (PedsQL 4.0, 2–4 years), and young children (PedsQL 4.0, 5–7 years) no (sufficiently reliable) Dutch normative data was available. For the PedsQL 4.0, normed within the Netherlands for ages 5–18 [15] and 18–30 years [16], no Dutch reference data existed for 2–4 year-old children and the reference sample of the 5–7 year old group was rather small.

Therefore, the aim of the current study was to provide Dutch reference data for the TAPQOL in infants aged 0–1 years and for the PedsQL 4.0 in toddlers aged 2–4 years, and in young children aged 5–7 years. Possible gender differences in the HRQoL of infants, toddlers, and young children were explored. Finally, we aimed to assess the internal consistency and to explore known-groups validity of the TAPQOL and PedsQL 4.0 in the concerning age groups.

## Methods

### Participants and procedures

The current study was part of a larger study [17] with the objective of establishing reliable and valid norm data for several parent-reported questionnaires about their children aged 0–18 years. Participants were recruited in November and December 2014.

Online data collection was carried out in collaboration with the Taylor Nelson Sofres Netherlands Institute for Public Opinion (TNS NIPO), a large Dutch market research agency. TNS NIPO provides access to respondents of TNS NIPObase. TNS NIPObase is a database with a panel of 200,000 respondents who have indicated that they are willing to participate in TNS NIPO research on a regular basis. TNS NIPO uses the software program ‘DIANA’ (www.niposoftware.com) for sampling and weighing procedures. This external agency was selected to gain a representative and large Dutch sample in a short period of time. The sample was stratified based on Dutch population figures regarding key demographics (age, sex, marital status and education). A stratified random sampling technique was used to minimize sample variance and increase precision. With the objective of obtaining around 1400 parents (expected response-rate of 60%), a total stratified sample of 2299 parents was drawn from the database. Inclusion criteria were: (1) one parent per family of a child aged 0–18 years, and (2) parents had to be able to read and understand a Dutch questionnaire. Parents received an invitational e-mail by TNS NIPO to participate in the panel. It was assumed that they could understand Dutch well enough once they had responded to the e-mail by registering themselves on the website. The current study aimed to establish norm data for the HRQoL measures in infants (0–1 years), toddlers (2–4 years), and young children (5–7 years). In total, 1305 parents out of the total stratified sample were invited that had a child between 0 and 7 years of age. These parents were included in the current study. Prior to the data-collection, TNS NIPO selected the age of the child per family that fitted with the age-specific versions of the questionnaires. While completing the questionnaires, parents were asked to keep in mind the selected child by TNS NIPO. Informed consent was obtained from all participants and the Medical Ethics Committee of the Academic Medical Center in Amsterdam, the Netherlands, approved of the study.

### Measures

#### Sociodemographics

Information regarding parental age, gender, educational level, country of birth, and employment (paid job) was provided by TNS NIPO for participants as well as non-participants. Educational level of the parent was classified according to the International Standard Classification of Education (ISCED, 2011): low = Level 0–2, medium = Level 3–5, high = Level 6–8. Participating parents indicated whether their child had a chronic condition: “Does your child have a chronic health condition? If Yes, please specify”. This was later categorized by a pediatrician in the Emma Children’s Hospital according to the criteria of Mokkink et al. [18].

#### TAPQOL

To collect Dutch parent-reported normative HRQoL data for children aged 0–1 years, the TNO-AZL Preschool Children Quality of Life (TAPQOL) questionnaire was used [8]. The 43 items (answer categories ‘never’, ‘sometimes’, ‘often’) are clustered into 12 multi-item scales, with higher scores (range 0–100) indicating better HRQoL: stomach (= gastrointestinal function), skin, lungs, sleeping, appetite, motor function, positive mood, anxiety, liveliness, problem behavior, social functioning and communication. The TAPQOL uses a 3-month recall period. The completion time is about 5–10 min and the overall psychometric properties are satisfactory [8].

#### PedsQL generic scale 4.0

To collect Dutch parent-reported normative HRQoL data for children aged 2–7 years, we used age-appropriate versions (age 2–4 years and 5–7 years) of the acute (1-week recall) generic Pediatric Quality of Life Inventory 4.0 (PedsQl 4.0) [1]. The PedsQL 4.0 contains 23 items, which are divided into a total scale score, a psychosocial domain (i.e., combined score of emotional, social, and school domain) and 4 subscales (5-point Likert scale): physical domain (8 items), emotional domain (5 items), social domain (5 items) and school domain (5 items). Higher scores indicated a better HRQoL (range 0–100).

### Statistical analyses

The Statistical Package for Social Sciences (SPSS) version 23.0 for Windows was used for all statistical analyses. First, differences in background characteristics between participants and non-participants were compared using a *t*-test (age) and Chi^2^-tests (gender, country of birth, educational level, and employment status).

Second, descriptives (median, mean, standard deviation) were used to display normative data on the TAPQOL for infants, the PedsQL 4.0 for toddlers, and the PedsQL 4.0 for young children. In addition, Mann–Whitney U tests were performed to determine a possible gender effect in the HRQoL of infants, toddlers, and young children.

Third, to determine internal consistency of the TAPQOL (0–1 years) and PedsQL 4.0 (2–4 years, and 5–7 years) domains, Cronbachs alpha coefficients were calculated based on the average inter-item correlation [19]. Cronbach’s alpha of < .60 was considered insufficient, .60–.69 as moderate, ≥.70 was regarded as satisfactory and ≥ .80 as good. Scales with reliabilities of .70 or greater are recommended for comparing patient groups, while Cronbach’s alphas of .90 are recommended for analyzing individual patient scale scores [20].

Finally, to study the known-groups validity, we conducted non-parametric Mann–Whitney U tests to compare TAPQOL and PedsQL 4.0 domain scores of healthy children with domain scores of children with a chronic health condition.

## Results

### Participants

In total, 794 parents participated (response rate 61%), including 50 (6.3%) parents of a child with a chronic condition. Reported chronic conditions in children aged 0–7 years were: eczema/skin conditions (18%), allergies (12%), skeletal or bone abnormality/cleft (12%), asthma/lung problems (8%), muscle disorder (8%), intellectual disability (6%) ADHD (6%), gastrointestinal disease (4%), autism/PDD-NOS (4%), heart disease (4%), chronic eye condition (4%), deaf or impaired hearing (4%), epilepsy (4%), kidney disease (4%), and haemophilia (2%).

The TAPQOL was completed by 227 parents of infants aged 0–1 years, including 9 parents (4%) of a child with a chronic disease. The toddler version of the PedsQL 4.0 (age category 2–4 years) was completed by 293 parents, of which 18 parents (6.1%) indicated to have a child with a chronic health condition. Two hundred and seventy-four parents completed the PedsQL 4.0 for young children (aged 5–7 years), of which 23 parents (8.4%) reported to have a child with a chronic health condition.

In Table 1, background characteristics of the participants and non-participants are presented for infants, toddlers, and young children separately. Participants and non-participants did not differ with regard to child gender, parental age, parental country of birth and parental employment status. However, significantly more mothers participated in the sample of young children, participants had a significant higher educational level in the toddler and young child sample, and children from participating parents in toddler sample were significantly younger than non-participating parents.

**Table 1:** Socio-demographic data of participants and non-participants, displayed separately for infants, toddlers and young children

	Participants	Non-participants	*p*
*Infant 0-1 years*	*M, SD,* % (*N* = 227)	*M, SD,* % (*N* = 86)	
Age	*0.98 (.52)*	*1.16 (.52)*	.006
Gender (male)	50.2	45.9	.495
*Parent*			
Age	33.95 (4.49)	34.06 (4.48)	.845
Gender (male)	41.9	41.9	.999
Country of birth (Netherlands)	97.4	96.5	.361
Educational level^a^			.045
Low	7.5	14.0	
Intermediate	28.2	38.4	
High	63.9	47.7	
Missing	0.3	-	
Paid employment (yes)	87.2	88.4	.774
*Toddler 2-4 years*	*M, SD,* % (*N* = 293)	*M, SD,* % (*N* = 200)	
Age	3.47 (.86)	3.56 (.84)	.250
Gender (male)	53.6	48.5	.267
*Parent*			
Age	36.60 (5.18)	35.99 (4.73)	.183
Gender (male)	39.6	43.5	.386
Country of birth (Netherlands)	94.2	96.5	.323
Educational level^a^			.606
Low	11.6	14.5	
Intermediate	44.0	41.5	
High	43.0	43.5	
Missing	1.4	0.5	
Paid employment (paid job: yes)	85.7	86.9	.689
*Young child 5-7 years*	*M, SD,* % (*N* = 274)	*M, SD,* % (*N* = 225)	
Age	6.52 (.87)	6.39 (.85)	.103
Gender (male)	55.6	54.7	.857
*Parent*			
Age	39.09 (5.32)	38.60 (5.18)	.229
Gender (male)	35.4	46.2	.014
Country of birth (Netherlands)	96.4	96.0	.408
Educational level^a^			.045
Low	10.6	17.8	
Intermediate	44.9	46.7	
High	44.2	34.7	
Missing	0.4	0.9	
Paid employment (yes)	85.3	81.3	.221

^a^ Highest educational level completed. Low: Primary education, lower vocational education, lower or middle general secondary education; Intermediate: middle vocational education, higher secondary education, pre-university education; High: higher vocational education, university

### Normative data for the TAPQOL and PedsQL 4.0

Table 2 presents the TAPQOL and PedsQL 4.0 mean and median scale scores of all participants per age category, and divided by children with and without a chronic health condition.

**Table 2:** Normative HRQoL data of Dutch infants, toddlers, and young children

		Total			Healthy			Chronic health condition			
	TapQoL subscales	N	Median	Mean (SD)	N	Median	Mean (SD)	N	Median	Mean (SD)	*p*
0-1 years old	Sleeping	227	75.00	75.9 (19.45)	218	81.25	76.23 (19.47)	9	75.00	68.05 (18.07)	.16
	Appetite	227	100.00	89.68 (15.65)	218	100.00	90.36 (15.27)	9	75.00	73.15 (16.55)	.001
	Lungs	227	100.00	92.88 (14.41)	218	100.00	93.08 (14.25)	9	100.00	87.96 (18.13)	.37
	Stomach	227	83.33	86.20 (15.92)	218	87.50	86.77 (15.00)	9	66.67	72.22 (28.87)	.20
	Skin	227	100.00	91.56 (12.11)	218	100.00	92.05 (11.61)	9	75.00	79.63 (17.73)	.013
	Motor functioning^a^	58	100.00	94.50 (12.72)	55	100.00	95.11 (11.52)	3	100.00	83.33 (28.87)	.62
	Social functioning^a^	58	100.00	92.53 (15.35)	55	100.00	93.03 (14.59)	3	100.00	83.33 (28.87)	.56
	Problem behavior	227	85.71	85.15 (14.63)	218	85.71	85.65 (14.63)	9	71.43	73.02 (8.58)	.003
	Communication^a^	58	100.00	88.47 (14.98)	55	100.00	88.41 (15.00)	3	100.00	89.58 (18.04)	.91
	Anxiety	227	100.00	86.27 (15.35)	218	100.00	86.47 (17.06)	9	83.33	81.48 (15.47)	.26
	Positive mood	227	100.00	98.24 (10.83)	218	100.00	98.17 (11.05)	9	100.00	100.00 (0.00)	.54
	Liveliness	227	100.00	97.65 (11.49)	218	100.00	97.71 (11.53)	9	100.00	96.30 (11.11)	.52
	PedsQL 4.0										
2-4 years old	Total score	293	90.48	88.55 (10.10)	275	90.48	89.16 (8.51)	18	88.69	79.23 (22.08)	.14
	Psychosocial domain	293	88.46	86.53 (11.14)	275	88.46	87.04 (10.02)	18	89.42	78.63 (21.12)	.31
	Physical domain	293	93.75	91.84 (11.22)	275	93.75	92.60 (9.26)	18	90.63	80.21 (25.07)	.016
	Emotional domain	293	80.00	78.45 (14.74)	275	80.00	78.78 (14.23)	18	77.50	73.33 (20.93)	.43
	Social domain	293	95.00	90.09 (13.71)	275	95.00	90.62 (12.91)	18	90.00	81.94 (21.70)	.14
	School domain	293	100.00	94.06 (12.49)	275	100.00	94.84 (10.35)	18	100.00	81.94 (28.04)	.04
	PedsQL 4.0										
5-7 years old	Total score	274	88.04	86.05 (11.59)	251	89.13	86.94 (10.85)	23	77.17	76.28 (14.83)	.001
	Psychosocial domain	274	86.67	83.38 (13.65)	251	88.33	84.40 (12.89)	23	70.00	72.17 (16.73)	.001
	Physical domain	274	93.75	91.06 (12.64)	251	96.88	91.71 (11.96)	23	90.63	83.97 (1728)	.013
	Emotional domain	274	80.00	77.93 (16.70)	251	80.00	78.78 (16.08)	23	70.00	68.48 (18.49)	.007
	Social domain	274	95.00	86.39 (16.70)	251	95.00	87.39 (16.17)	23	80.00	75.43 (18.82)	.001
	School domain	274	90.00	85.82 (15.41)	251	90.00	87.03 (14.33)	23	75.00	72.61 (20.27)	.001

^a^ TAPQOL domain only contains items for children aged ≥ 1,5 years

### Gender differences in TAPQOL and PedsQL 4.0 scores

Table 3 shows normative scores for the total infant, toddler, and young child sample, with scores displayed separately for boys and girls. No differences in HRQoL scores were found between boys and girls, except for the ‘problem behavior’ scale of the TAPQOL, which showed less behavioral problems for infant girls than for infant boys (Table 3).

**Table 3:** Normative HRQoL data of total sample of Dutch infants, toddlers, and young children, displayed separately for boys and girls

		Boys			Girls			
	TapQoL subscales	N	Median	Mean (SD)	N	Median	Mean (SD)	*p*
0-1 years old	Sleeping	114	75.00	73.68 (20.70)	113	81.25	78.15 (17.92)	.148
	Appetite	114	100.00	89.69 (16.01)	113	100.00	89.68 (15.28)	.623
	Lungs	114	100.00	92.54 (14.93)	113	100.00	93.22 (13.93)	.952
	Stomach	114	91.67	87.57 (15.21)	113	83.33	84.81 (16.56)	.163
	Skin	114	91.67	90.35 (12.42)	113	100.00	92.77 (11.71)	.082
	Motor functioning^a^	28	100.00	92.63 (14.93)	30	100.00	96.25 (10.20)	.368
	Social functioning^a^	28	100.00	92.86 (15.33)	30	100.00	92.22 (15.62)	.708
	Problem behavior	114	85.71	82.77 (15.49)	113	92.86	87.55 (13.36)	.013
	Communication^a^	28	87.50	85.49 (17.76)	30	100.00	91.25 (11.44)	.285
	Anxiety	114	83.33	85.09 (17.23)	113	100.00	87.46 (16.75)	.233
	Positive mood	114	100.00	98.54 (10.05)	113	100.00	97.94 (11.60)	.718
	Liveliness	114	100.00	97.95 (11.12)	113	100.00	97.35 (11.90)	.567
	PedsQL 4.0							
2-4 years old	Total score	157	90.48	87.78 (10.64)	136	91.67	89.44 (9.41)	.172
	Psychosocial domain	157	88.46	85.68 (11.35)	136	88.46	87.50 (10.86)	.132
	Physical domain	157	93.75	91.18 (12.37)	136	93.75	92.60 (9.73)	.380
	Emotional domain	157	80.00	78.10 (14.15)	136	80.00	78.86 (15.43)	.521
	Social domain	157	95.00	88.92 (14.34)	136	100.00	91.43 (12.88)	.083
	School domain	157	100.00	92.94 (14.06)	136	100.00	95.34 (10.28)	.095
	PedsQL 4.0							
5-7 years old	Total score	152	86.96	85.56 (11.47)	122	90.22	86.65 (11.77)	.277
	Psychosocial domain	152	85.00	82.34 (13.83)	122	88.33	84.67 (13.37)	.120
	Physical domain	152	93.75	91.61 (11.11)	122	96.88	90.37 (14.34)	.859
	Emotional domain	152	80.00	76.84 (16.17)	122	80.00	79.26 (16.88)	.146
	Social domain	152	90.00	85.53 (16.98)	122	95.00	87.46 (16.35)	.207
	School domain	152	90.00	84.64 (15.51)	122	95.00	87.30 (15.21)	.071

^a^TAPQOL domain only contains items for children aged ≥ 1,5 years

### Internal consistency of the TAPQOL and PedsQL 4.0

Due to the small number of children with a chronic health condition, it was not possible to conduct separate reliability analyses for the group of healthy children and children with a chronic condition. Table 4 presents the Cronbach’s alphas of all participants within each age group (i.e., 0–1 years, 2–4 years, and 5–7 years). All TAPQOL scales had moderate to good reliability (α = .60–.92), except for the ‘stomach’ scale (α = .39). Regarding the PedsQL 4.0 for toddlers, the total scale and all subscales reached moderate to good reliability (α = .60–.88). For young children (5–7 years), all PedsQL 4.0 total scales and subscales reached satisfactory to excellent reliability (α = .76–.90).

**Table 4:** Internal consistency (Cronbach’s alpha) of the (sub) scales

			Total sample (*N* = 794)	
	TAPQOL subscales	Number of items	N	α
0–1 years old	Sleeping	4	227	.85
	Appetite	3	227	.80
	Lungs	3	227	.68
	Stomach	3	227	.39^b^
	Skin	3	227	.73
	Motor functioning^a^	4	58	.90
	Social functioning^a^	3	58	.76
	Problem behavior	7	227	.78
	Communication^a^	4	58	.88
	Anxiety	3	227	.60
	Positive mood	3	227	.92
	Liveliness	3	227	.88
	PedsQL 4.0			
2–4 years old	Total score	21	293	.88
	Psychosocial health	13	293	.84
	Physical health	8	293	.80
	Emotional functioning	5	293	.78
	Social functioning	5	293	.78
	School functioning	3	293	.60
	PedsQL 4.0			
5–7 years old	Total score	23	274	.90
	Physical health	8	274	.82
	Psychosocial health	15	274	.89
	Emotional functioning	5	274	.81
	Social functioning	5	274	.82
	School functioning	5	274	.76

α Cronbach’s coefficient alpha

^a^TAPQOL domain only contains items for children aged ≥ 1,5 years

^b^ *a* < .60 are considered insufficient

### Known-groups validity of the TAPQOL and PedsQL 4.0

Infants (0–1 years) with a chronic health condition had significantly lower HRQoL scores on 3 out of 12 scales of the TAPQOL (i.e., ‘appetite’, ‘skin’, and ‘problem behavior’), compared to healthy children. Toddlers (2–4 years) with a chronic health condition had significantly lower parent-reported HRQoL scores compared to healthy children on 2 out of 6 PedsQL 4.0 domains (‘physical’ and ‘school functioning’). For young children (5–7 years) with a chronic health condition, parents reported a significant lower HRQoL on all PedsQL 4.0 domains (i.e., ‘total’, ‘psychosocial’, ‘physical’, ‘emotional’, ‘social’, and ‘school’) compared to children without a chronic health condition.

## Discussion

The current study aimed to provide Dutch HRQoL normative data for the TAPQOL for infants (aged 0-1 years), the PedsQL 4.0 for toddlers (aged 2–4 years), and the PedsQL 4.0 for young children (aged 5–7 years). Furthermore, we examined the influence of gender on parent-reported HRQoL and we assessed the internal consistency and known-groups validity of the TAPQOL and PedsQL 4.0 in the concerning age groups.

Overall, no gender effect was found in the HRQoL of infants, toddlers, and young children. Yet, infant girls had less behavioral problems than infant boys on the TAPQOL ‘problem behavior scale’. This in line with a study that looked at sex differences in children up to 8 years of age, where parents reported that infant girls showed less behavioral problems than infant boys [21].

Moderate to good reliability was found for all TAPQOL domains, except for the TAPQOL ‘stomach’ scale (α = .39). The original study conducted by Fekkes et al. [8] on the psychometric properties of the TAPQOL in 1–5 year-old healthy children, also found a rather weak reliability for the ‘stomach’ scale (α = .51). The items included in this scale measure cramps, stomach or belly pain, and nausea. These items do not have to necessarily correlate with each other, which might have accounted for the low reliability we found for this scale. For example, infants experiencing cramps do not have to be nauseous per definition. Thus, cautiousness is warranted with using this scale to measure stomach problems in infants.

In our sample of toddlers aged 2–4 years, all PedsQL 4.0 scales reached moderate to good reliability. The lowest Cronbach’s alpha was found for the PedsQL 4.0 ‘school functioning’ domain (α = .60). Varni, Limbers, and Burwinkle [22] also found the lowest alpha for the PedsQL 4.0 ‘school functioning’ domain in the 2–4 year-old age group (α = .59–.69). Relatively lower Cronbach’s alphas might be related to the small number of items included in a scale [22, 23]. In the 5–7 year-old age group, the PedsQL 4.0 had satisfactory to good reliability for all domains. Across the different age groups, Cronbach’s alphas seemed to increase slightly with the child’s age. Similar results were found in a study comparing children’s proxy-reported HRQoL across different age groups, using the PedsQL 4.0 [22]. In general, this study showed that the TAPQOL and PedsQL 4.0 are reliable instruments to measure HRQoL in infants, toddlers and young children in the Dutch normative population. Scales with lower reliability coefficients should only be used for explorative purposes [22].

Relating to the known-groups validity, for young children (aged 5–7 years), differences in scores between healthy and chronically ill children were found on all PedsQL 4.0 domains, indicating the PedsQL 4.0 to be a valid instrument to distinguish HRQoL in healthy children and in children with a chronic condition in this age category. The TAPQOL only differentiated between children with and without a chronic health condition in 3 out of 12 domains. The initial study performed by Fekkes et al. [8] among children 1–5 years also found that several scales of the TAPQOL did not differentiate between children with and without a chronic health condition. Toddlers with a chronic health condition differed from healthy toddlers on 2 out of 6 domains on the PedsQL 4.0. The study conducted by Varni, Limble and Burwinkle [22] found significant differences for all PedsQL 4.0 domains in this age group. Yet, analyses in their study were conducted on a larger sample (*N* = 617) of toddlers with a chronic health condition, which might have enabled them to find significant differences. It is therefore the question whether, in very young children (i.e., infants or toddlers), a valid difference can be made in HRQoL between children with and without a chronic health condition. Despite the fact that average HRQoL scores may not always differentiate between children with and without a chronic health condition, poor HRQoL scores may still provide clinically meaningful information [24].

This study has some limitations to note. First, even though we did our best to deliver a sample that was as representative for the general Dutch population as possible, participants and non-participants still slightly differed with regard to the child’s age, parental gender, and parental educational level. It is widely known that women and highly educated people are often more willing to participate in research. Second, as mentioned earlier, the sample size of children with a chronic health condition in the sample of infants and toddlers was small and it might have been the case that we did not have enough power to be able to find significant differences between children with and without a chronic health condition. Future studies should include larger age subgroups of infants and toddlers with a chronic health condition, such that can be sufficiently determined whether the TAPQOL and PedsQL 4.0 differentiate HRQoL in children with and without a chronic health condition. Third, parents judged themselves whether their child had a chronic health condition. It could therefore be the case that our sample reflected a slightly different group of children with a chronic condition than when this would have been directly diagnosed by a physician. Finally, since this study was related to a larger project on the implementation of patient-reported outcomes in clinical practice [14], we chose to provide Dutch normative data for brief infant, toddler, and young child electronic HRQoL questionnaires that were already available in Dutch and in use by our hospital at that time. Since 94% of households in the Netherlands have access to the internet, we think it is safe to say that using electronic measures did not have a tremendous impact on the generalizability of the findings [25]. Yet, other possible reliable and valid paper-pencil HRQoL measures (e.g., the PedsQL 4.0 infants scales [24], or the Infant and Toddler Quality of Life questionnaire [7]) are worth studying as well. Future research could determine which questionnaires are best in assessing HRQoL in infants and toddlers.

## Conclusion

In conclusion, our study indicates that for children aged 0–4 years, HRQoL can be reliably measured with the TAPQOL and the PedsQL 4.0, but it remains unclear whether these HRQoL instruments can distinguish between infants and toddlers with and without a chronic health condition. The findings of the current study support the internal consistency of the PedsQL 4.0 for children aged 5–7 years of age.
